# Expression Patterns of LALBA and Nucleolin and Their Clinical, Prognostic, and Immune Relevance in Breast Cancer Tissues of Mexican Patients

**DOI:** 10.3390/ijms27031561

**Published:** 2026-02-05

**Authors:** Mariana Navarro-Real, Juan Omar Zavala-López, Juliana Marisol Godínez-Rubí, Antonio Quintero-Ramos, Alicia Del Toro-Arreola, Ramon Franco-Topete, Ángel Quiroz Bolaños, Antonio Topete, Adrián Daneri-Navarro

**Affiliations:** 1 Instituto de Investigación en Inmunología, Departamento de Fisiología, Centro Universitario de Ciencias de la Salud (CUCS), Universidad de Guadalajara, Guadalajara 44340, Mexico; mariana.navarro@alumnos.udg.mx (M.N.-R.); antonio.qramos@academicos.udg.mx (A.Q.-R.); alicia.deltoro@academicos.udg.mx (A.D.T.-A.); angelquirozqfb@gmail.com (Á.Q.B.); 2 Laboratorio de Bioquímica Estructural, Departamento de Química, Centro Universitario de Ciencias Exactas e Ingeniería (CUCEI), Universidad de Guadalajara, Guadalajara 44430, Mexico; juan.zavala@alumnos.udg.mx; 3 Laboratorio de Patología Diagnóstica e Inmunohistoquímica (LAPADI), Departamento de Microbiología y Patología, Centro Universitario de Ciencias de la Salud (CUCS), Universidad de Guadalajara, Guadalajara 44340, Mexico; juliana.godinez@academicos.udg.mx (J.M.G.-R.); ramon.ftopete@academicos.udg.mx (R.F.-T.); 4 Colloids and Polymers Physics Group, Particle Physics Department, Materials Institute (iMATUS) and Health Research Institute (IDIS), University of Santiago de Compostela, 15782 Santiago de Compostela, Spain

**Keywords:** breast cancer, LALBA, nucleolin, TILs, PD-L1, Tim-3

## Abstract

Breast cancer is the most common and deadliest cancer among women. While overexpression of specific markers guides disease stratification and has enabled the development of targeted therapies, identifying new therapeutic targets remains critical, particularly for aggressive subtypes lacking effective treatments. This study evaluated the expression of α-Lactalbumin (LALBA) and nucleolin (NCL) in breast cancer tissues from Mexican patients using gene expression analysis and immunohistochemistry. LALBA, a major milk protein normally expressed only during late pregnancy and lactation, was detected in nearly all tumor samples and showed higher levels in aggressive subtypes, with overexpression displaying a slight trend toward poorer overall survival. NCL, a multifunctional nucleolar protein, exhibited predominantly nuclear localization, with moderate expression associated with improved survival. Both proteins correlated with tumor immune features, including increased tumor-infiltrating lymphocytes (TILs) and PD-L1 expression for LALBA, and elevated CD8^+^ T cells, PD-L1, and TIM-3 expression for NCL. Overall, these findings suggest that LALBA and NCL are associated with tumor aggressiveness, immune context, and survival trends in breast cancer. Additional studies in larger cohorts are needed to define their clinical relevance.

## 1. Introduction

Breast cancer is the most common type of cancer in women. According to statistical data from the World Health Organization, in 2022, breast cancer represented the first leading cause of global female cancer incidence, with an estimated 2.3 million new cases, and it was also the first leading cause of female cancer mortality, with 665,684 deaths [1]. Breast cancer is a multidimensional, complex, and highly heterogeneous disease, encompassing multiple tumor subtypes, each characterized by a molecular signature and with a variety of morphological patterns [2]. Regarding molecular heterogeneity, breast cancer is generally classified into five main molecular subtypes: luminal A, luminal B (HER2−), luminal B (HER2+), HER2-enriched, and Triple-Negative (TN). Although comprehensive molecular analysis is considered the gold standard, in routine clinical practice, this classification is typically operationalized through immunohistochemical evaluation of four markers: estrogen receptor (ER), progesterone receptor (PR), human epidermal growth factor receptor 2 (HER2), and Ki-67, as described in Table 1 [3].

The overexpressed receptors evaluated by immunohistochemistry (IHC) in breast cancer—ER, PR, HER2—not only facilitate cancer classification, but they also serve as prognostic markers and constitute potential targets for anticancer therapy [4]. In fact, the approval of Trastuzumab, the first anti-HER-2 monoclonal antibody, revolutionized breast cancer therapy [5] and paved the way for the identification of several new specific markers, known as tumor antigens, which are candidates to become therapeutic targets [6]. Nowadays, other receptors overexpressed in cancer tissues—known as tumor associated antigens (TAAs)—such as glycoprotein non-metastatic melanoma protein b (GpNMB) [7], trophoblast cell-surface antigen 2 (Trop-2) [8] and protein tyrosine kinase 7 (PTK7) receptor [9] have been successfully targeted with promising clinical outcomes. Despite these advances, identifying novel tumor antigens remains critical to enable the development of more precise and personalized treatments, allow overcome resistance mechanisms, and improve patient prognosis, especially in cancer subtypes that still lack an effective targeted therapy [10].

In this regard, α-Lactalbumin (LALBA) has emerged as a potential TAA in breast cancer therapy. In 2016, Tuohy et al. observed the overexpression of LALBA in 8 of 11 Triple-Negative breast cancer biopsies [11]. LALBA is a 16.2 kDa metalloprotein expressed exclusively in the mammary parenchyma and, under normal physiological conditions, only during the third trimester of pregnancy and lactation, functioning as an enzymatic modulator of lactose synthase. The expression of LALBA in the mammary epithelium is tightly regulated, with its transcription influenced by the presence of prolactin and insulin and inhibited by progesterone [12,13,14].

Another potential TAA candidate is nucleolin (NCL), a nucleolar phosphoprotein that predominantly localized in the nucleolus (approximately 90%) but is also present in the nucleoplasm, cytoplasm, and on the cell surface. Within the nucleolus, NCL functions during the early stages of ribosome biogenesis, regulating rDNA chromatin organization, RNA polymerase I transcription, pre-rRNA processing, and pre-ribosome assembly. Although NCL is expressed in both normal and malignant tissues, its expression is significantly higher in breast cancer, where it promotes tumor cell proliferation and angiogenesis [15,16].

Despite their distinct biological functions and cellular localization, both LALBA and NCL have been proposed as tumor-associated antigens in breast cancer and linked to more aggressive tumor phenotypes [11,14,15]. However, they have not been jointly examined within the same patient cohort. A parallel evaluation of these markers may therefore offer complementary insights into tumor heterogeneity and immune contexture.

Based on these considerations, we exploratorily assessed LALBA and NCL expression in breast cancer tissues from patients in Western Mexico to examine their associations with clinicopathological characteristics, overall survival, and immune-related markers. We hypothesized that the expression patterns of these markers may be linked to differences in tumor aggressiveness and immune microenvironment across molecular subtypes. Given the limited and sometimes conflicting evidence in the literature, this study was designed as hypothesis-generating rather than to establish causal or prognostic conclusions.

## 2. Results

### 2.1. Description of the Cohort Analyzed in This Study

Table 2 summarizes the clinical and pathological information of the patients included in this study. The tumor subtype frequencies were luminal A (*n* = 21, 32.3%), triple-negative (*n* = 18, 27.7%), luminal B (*n* = 14, 21.5%), and HER2-enriched subtype (*n* = 12, 18.5%). Most patients included in this study were over 51 years old (*n* = 38, 58.5%), had invasive ductal carcinoma (*n* = 49, 75.4%), and an intermediate histological grade (*n* = 36, 55.4%). Usually, the patients were diagnosed at stage II (*n* = 28, 43.1%) or stage III (*n* = 25, 38.5%).

### 2.2. LALBA and NCL Gene Expression in Breast Cancer Tissues

**Figure 1:**
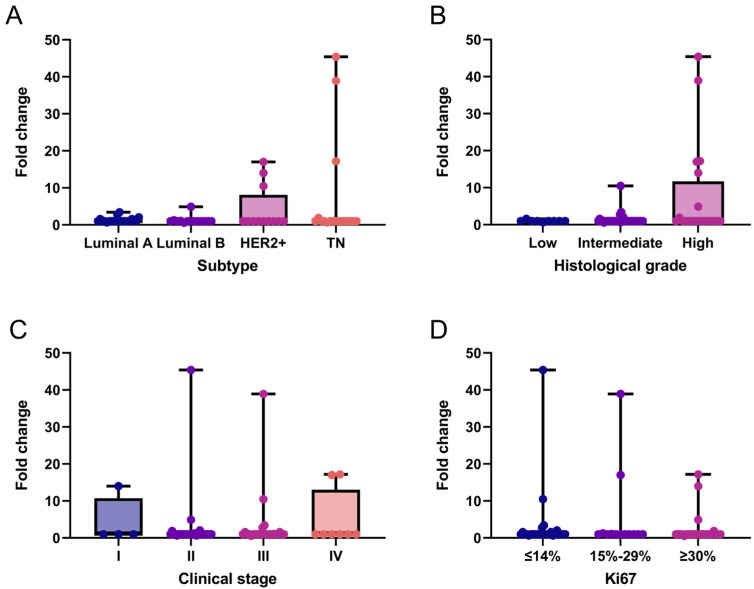
*LALBA* gene expression patterns in breast cancer tissues. *LALBA* mRNA expression across molecular subtypes (**A**), histological grades (**B**), clinical stages (**C**), and Ki67 proliferation index (**D**). Box-and-whisker plot showing individual data points. Boxes represent the interquartile range (Q1–Q3) with the median line; whiskers extend to the minimum and maximum values. *n* = 65. No statistically significant differences between categories were observed (Kruskal–Wallis test). TN: Triple-Negative.

We performed an exploratory analysis of *LALBA* gene expression in breast cancer tissues and descriptively evaluated its distribution across patients’ clinicopathological characteristics. Overall, *LALBA* expression was generally low across breast cancer subtypes, with 76.9% (*n* = 50) of patients exhibiting fold-change values ≤ 1. In contrast, 13.8% (*n* = 9) of patients showed moderate expression levels (fold-change between 1 and 5), and only 9.2% (*n* = 6) exhibited markedly high expression (fold-change > 5). These high-expression cases were found exclusively in the HER2+ and triple-negative subtypes (Figure 1A), which also corresponded to intermediate to high histological grades in the analyzed cohort (Figure 1B). No statistically significant associations was observed between *LALBA* expression and clinical stage (Figure 1C) or Ki67 levels (Figure 1D).

**Figure 2:**
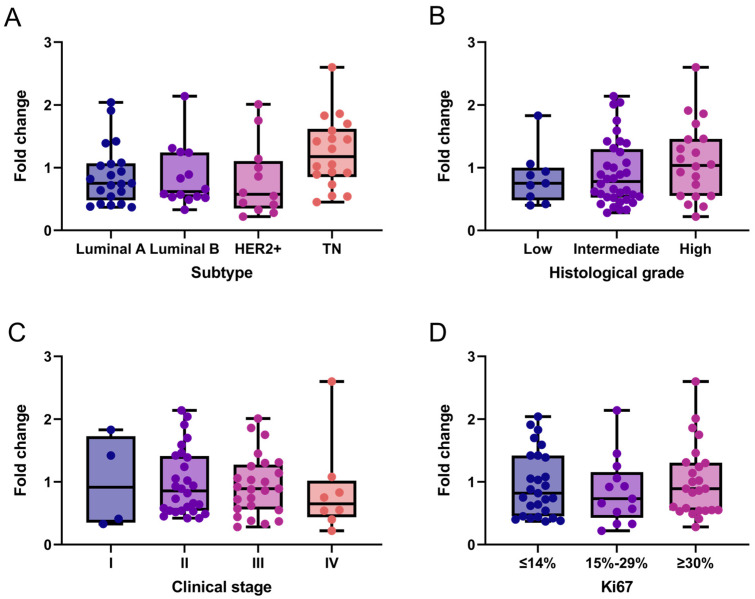
*NCL* gene expression patterns in breast cancer tissues. *NCL* mRNA expression across molecular subtypes (**A**), histological grades (**B**), clinical stages (**C**), and Ki67 proliferation index (**D**). Box-and-whisker plot showing individual data points. Boxes represent the interquartile range (Q1–Q3) with the median line; whiskers extend to the minimum and maximum values. *n* = 65. No statistically significant differences between categories were observed (Kruskal–Wallis test). TN: Triple-Negative.

Additionally, we exploratorily assessed *NCL* mRNA expression in the same breast cancer tissue samples. Overall, *NCL* levels expression were low across all subtypes. Specifically, 61.5% (*n* = 40) of patients exhibited low *NCL* expression (fold-change ≤ 1), while the remaining 38.5% (*n* = 25) showed moderate expression levels ranging from 1 to 2.6. A slight trend toward increased *NCL* expression was observed in triple-negative breast cancer (Figure 2A) and in tumors with higher histological grade (Figure 2B), which were predominantly HER2+ and triple-negative. Although no clear association was found between *NCL* expression and breast cancer stages, 84.6% (22 of 26) of samples with fold-change values > 1 were observed in stages II and III compared to stage IV (Figure 2C). In contrast, no association was observed between *NCL* expression and the Ki67 proliferation marker (Figure 2D).

### 2.3. Survival Analysis of Breast Cancer Patients According to LALBA and NCL mRNA Expression

**Figure 3:**
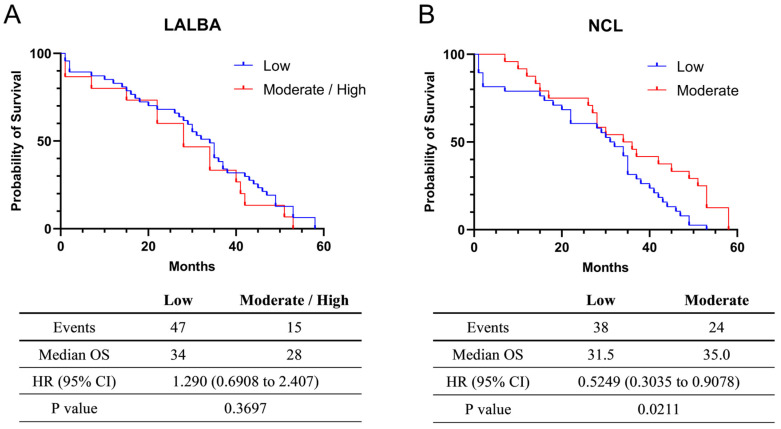
Survival analysis in Kaplan–Meier plotter. Relationship between *LALBA* (**A**) and *NCL* (**B**) mRNA expression and the survival of breast cancer patients.

The exploratory univariate analysis of overall survival (OS) was 34 months for the 47 patients with low (fold-change values ≤ 1) *LALBA* expression and 28 months for the 15 patients with moderate/high (fold-change values > 1) *LALBA* expression. Although patients with moderate/high *LALBA* expression appeared to have shorter median survival than those with low expression (Figure 3A), this effect did not reach statistical significance (Kaplan–Meier survival curves, *p* > 0.05). Additionally, a univariate Cox proportional hazards model yielded a hazard ratio (HR) of 1.290, suggesting a higher risk of death in the moderate/high expression group. However, the 95% confidence interval (CI: 0.6908–2.407) encompassed 1, indicating that the survival difference between the moderate/high and low *LALBA* expression groups was not statistically significant (*p* = 0.3697).

For *NCL* mRNA expression, the exploratory univariate survival analysis indicated a median OS of 31.5 months among the 38 patients with low expression (fold-change values ≤ 1) and 35.0 months among the 24 patients with moderate expression (fold-change values > 1). These findings suggest that moderate *NCL* expression is associated with improved median survival (Figure 3B). Consistent with this observation, the hazard ratio was 0.5249, indicating a lower risk of death in the moderate-expression group. Because the 95% confidence interval (CI = 0.3035–0.9078) did not include 1, the survival difference between the moderate and low *NCL* expression groups was statistically significant (*p* = 0.0211). Nevertheless, these results should be interpreted with caution due to the limited sample size and unadjusted analysis. Information on treatment regimens and standardized follow-up duration was not available for all patients and was therefore excluded from these survival analyses.

### 2.4. LALBA and NCL Protein Expression in Breast Cancer Biopsies

**Figure 4:**
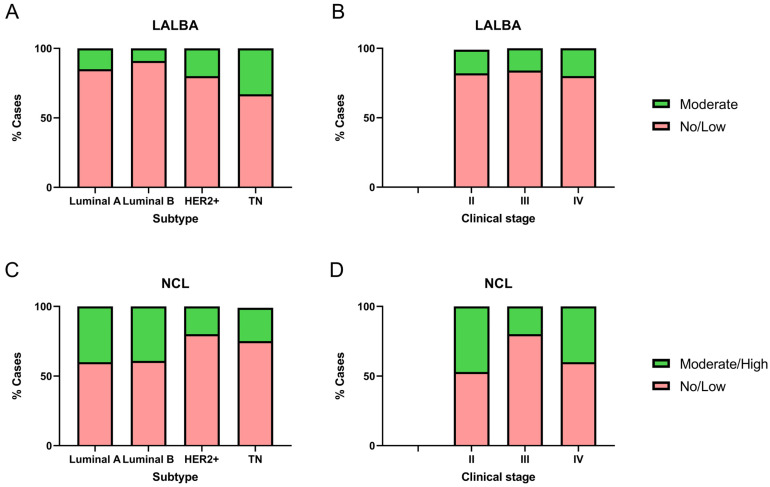
Immunohistochemical detection of LALBA and NCL in patient biopsies. LALBA and NCL expression by IHC in breast cancer biopsies across molecular subtypes (**A**,**C**) and clinical stages (**B**,**D**). Both markers showed significant differences in expression among molecular subtypes. No significant differences were observed in LALBA expression with respect to clinical stage; however, a statistically significant association was found between clinical stage and NCL immunohistochemical expression (Chi-square and Fisher’s exact tests).

We analyzed LALBA and NCL expression levels in 51 breast cancer biopsy samples and evaluated their association with molecular subtypes and clinical stages (Figure 4). Notably, none of the biopsies corresponded to stage I, and four samples lacked sufficient tumor tissue, which prevented the identification of LALBA in the tumor area. It is essential to note that none of the available biopsies matched the samples with high LALBA mRNA expression. Of the 47 biopsies available for LALBA expression evaluation, 42.5% (*n* = 20) were Luminal A, 23.4% (*n* = 11) Luminal B, 21.3% (*n* = 10) HER2+, and 12.8% (*n* = 6) Triple-Negative. Of those, 83.0% (*n* = 39) showed no or faint staining, and the remaining 17.0% (*n* = 8) exhibited moderate expression. To further assess the relationship between LALBA mRNA and protein expression, we performed Pearson and Spearman correlation analyses between mRNA fold-change values and IHC scores. Pearson correlation showed a moderate but significant association (r = 0.39, *p* = 0.0107), whereas Spearman correlation was not statistically significant (r = 0.24, *p* = 0.1326). These results indicate partial concordance but also highlight substantial mRNA–protein discordance, which aligns with known post-transcriptional regulatory variability in cancer tissues.

Among the eight samples showing 2+ or moderate LALBA expression, three were Luminal A, one was Luminal B, two were HER2+, and two were Triple-Negative (Figure 4A). The marker expression differed significantly among breast cancer subtypes (χ^2^ = 20.12, df = 3, *p* = 0.0002). Post hoc pairwise comparisons using Fisher’s exact test showed that Triple-Negative tumors had a significantly higher proportion of moderate expression compared to Luminal A (*p* = 0.0046) and Luminal B (*p* < 0.0001), while HER2+ tumors also exhibited a significantly higher proportion of moderate expression compared to Luminal B (*p* = 0.0432). These results indicate that moderate LALBA expression was predominantly detected in the more aggressive subtypes, HER2+ and Triple-Negative.

Given this distribution, subtype represents a potential confounding factor in the interpretation of LALBA protein expression. Although the sample size did not allow formal subtype-stratified correlation tests, we acknowledge this limitation and explicitly note that mRNA–protein concordance may be influenced by tumor subtype composition. No significant differences were observed with respect to clinical stage (χ^2^ = 0.58, df = 2, *p* = 0.7484; Figure 4B).

Of the 51 biopsies available for NCL protein evaluation, 39.2% (*n* = 20) were Luminal A, 25.5% (*n* = 13) Luminal B, 19.6% (*n* = 10) HER2+, and 15.7% (*n* = 8) Triple-Negative. Among these, 66.7% (*n* = 34) showed no or faint staining, whereas 33.3% (*n* = 17) exhibited moderate to high expression. Within the group with moderate/high expression, eight samples were Luminal A, five Luminal B, two HER2+, and two Triple-Negative (Figure 4C). Marker expression differed significantly among breast cancer subtypes (χ^2^ = 14.59, df = 3, *p* = 0.0022). Post hoc pairwise comparisons using Fisher’s exact test showed that Triple-Negative tumors had a significantly lower proportion of moderate/high expression compared to Luminal A (*p* = 0.0226) and Luminal B (*p* = 0.0327). Similarly, HER2+ tumors also exhibited a significantly lower proportion of moderate/high expression compared to both Luminal A (*p* = 0.0032) and Luminal B (*p* = 0.0050).

To examine the relationship between transcript and protein abundance, we evaluated the association between NCL mRNA fold-change values and IHC scores. Pearson (r = 0.28, *p* = 0.1532) and Spearman (r = 0.27, *p* = 0.1679) coefficients indicated weak and non-significant correlations, reflecting limited concordance between mRNA and protein expression. This modest association suggests the presence of mRNA–protein discordance, a phenomenon frequently observed in cancer tissues due to post-transcriptional regulation, differential protein stability, or technical variability between detection platforms.

Although a statistically significant association was observed between clinical stage and NCL immunohistochemical expression (χ^2^ = 17.11, df = 2, *p* = 0.0002; Figure 4D), all cases with moderate/high expression in stages III and IV, as well as the majority in stage II, correspond to Luminal A or Luminal B. This pattern suggests that the subtype may be more determinant than the stage. This distribution pattern indicates that molecular subtype may exert a stronger influence on NCL expression than clinical stage. Given the limited sample size within individual categories, subtype-stratified correlation analyses were not feasible, which represents a limitation of the present study. Overall, these results indicate that moderate/high NCL expression was predominantly detected in the less aggressive subtypes, Luminal A and Luminal B, consistent with the Kaplan–Meier survival analysis.

**Figure 5:**
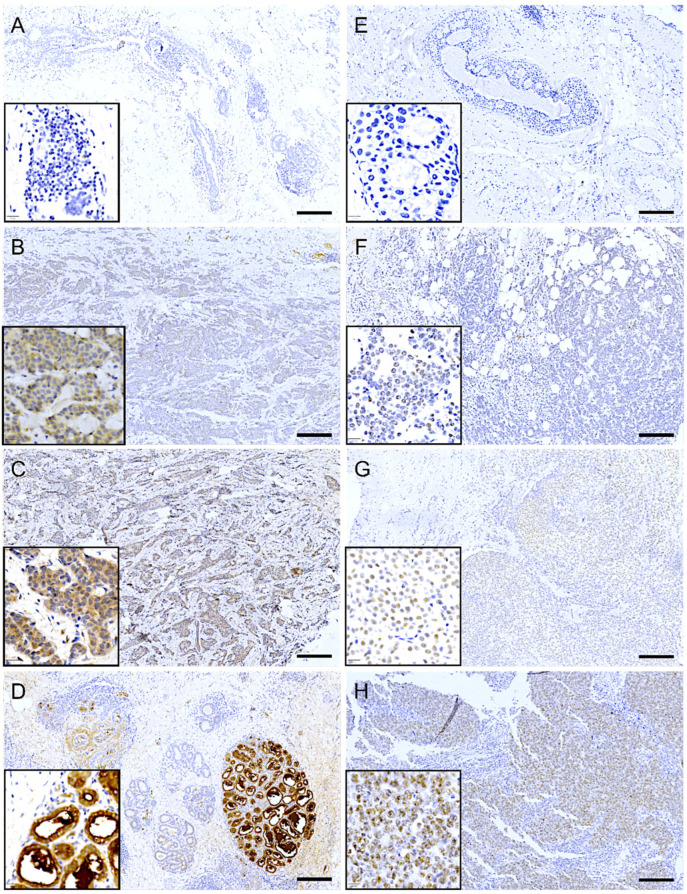
Immunohistochemical expression of LALBA and NCL in patient biopsies. Representative images of breast cancer tissues analyzed for LALBA expression by immunohistochemistry, showing a negative sample (**A**), as well as samples with low (**B**) and moderate (**C**) expression. High expression is observed in a healthy lobule (**D**). Representative images of breast cancer tissues stained for NCL, showing a negative sample (**E**), as well as samples with low (**F**), moderate (**G**), and high (**H**) expression. Inset, area of 20× amplification. Scale bar = 200 µm.

Immunohistochemical analysis revealed that LALBA expression in tumor cells was predominantly cytoplasmic. In most biopsies with positive staining, the expression pattern was focal or multifocal, involving approximately 10% to 70% of the tumor tissue. The staining intensity in these cases ranged from low (Figure 5B) to moderate (Figure 5C). Notably, in approximately 20% of the positive cases, the expression pattern was diffuse, extending to 70–100% of the tissue, albeit often with discrete focal areas of higher intensity. Importantly, high (3+) expression was not observed in tumor tissue but was exclusively detected in adjacent normal mammary lobules (Figure 5D).

In contrast, immunohistochemical analysis of NCL revealed a predominantly nuclear localization pattern in tumor cells. Among the biopsies with positive staining, most exhibited a diffuse expression pattern, involving approximately 40% to 90% of the tumor tissue. The staining intensity varied across samples, ranging from low to high (Figure 5F–H). Interestingly, approximately 40% of the positive cases displayed a focal or multifocal expression pattern, with staining confined to 10% to 40% of the tumoral tissue.

### 2.5. Association of LALBA and NCL Gene Expression with Immune-Related Markers in Breast Tumors

**Figure 6:**
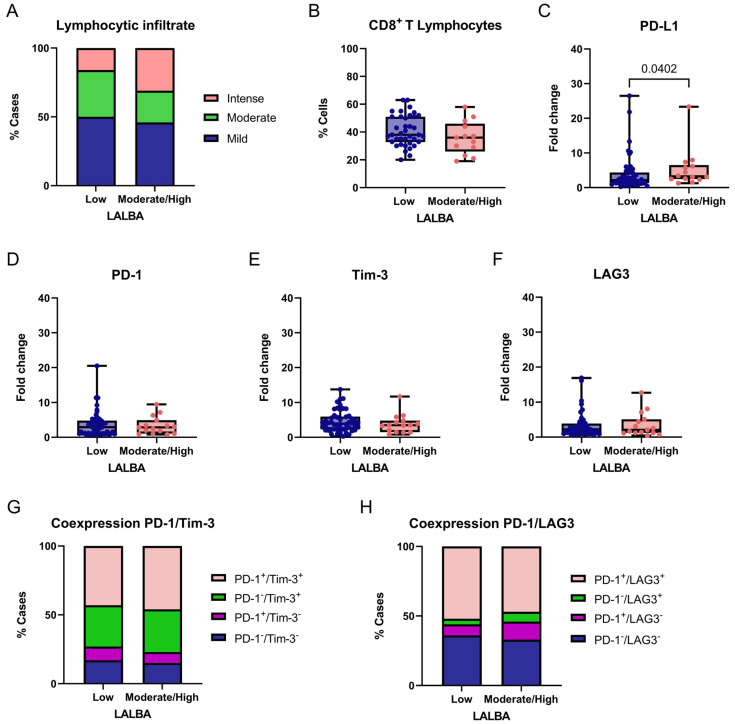
Association between *LALBA* expression and immune-related markers in breast tumors. Comparison of degree of lymphocytic infiltration (**A**), percentage of CD8^+^ T cells (**B**), PD-L1 (**C**), PD-1 (**D**), Tim-3 (**E**), and LAG3 (**F**) expression levels, and PD-1/Tim-3 or PD-1/LAG3 coexpression (**G**,**H**) according to *LALBA* expression groups (Low vs. Moderate/High). Box-and-whisker plot showing individual data points. Boxes represent the interquartile range (Q1–Q3) with the median line; whiskers extend to the minimum and maximum values. *n* = 65. Statistically significant differences were observed in the lymphocytic infiltrate and PD-L1 expression (Chi-square and Fisher’s exact tests or Mann–Whitney U test).

We exploratorily and descriptively compared *LALBA* low gene expression (fold-change values ≤ 1) and moderate/high gene expression (fold-change values > 1) with immune-related markers in breast tumors. Given the exploratory design, no correction for multiple testing was applied, and results should be interpreted cautiously. Lymphocytic infiltration differed significantly among *LALBA* expression (χ^2^ = 7.077, df = 2, *p* = 0.0291; Figure 6A). Post hoc Fisher’s exact tests indicated that this difference was primarily driven by a higher frequency of intense lymphocytic infiltration in tumors with moderate/high *LALBA* expression compared to tumors with low *LALBA* expression (31% vs. 16%; *p* = 0.0190).

No significant differences were observed for the mild infiltration (50% vs. 46%, *p* = 0.6712) or moderate infiltration (34% vs. 23%, *p* = 0.1168). These results suggest a potential association between higher *LALBA* expression and increased intense lymphocytic infiltration; however, no functional immune assays were performed, and no prognostic or mechanistic inferences can be drawn from these observations. The proportion of CD8^+^ T cells within the lymphocytic infiltrate was compared between tumors with low and moderate/high *LALBA* expression (Figure 6B). No significant difference was observed between the groups, indicating that while *LALBA* expression may influence the overall intensity of lymphocytic infiltration, it does not appear to specifically affect the relative abundance of CD8^+^ T cells within the infiltrate.

We then evaluated the expression of the immune checkpoint markers PD-L1, PD-1, and TIM-3 between tumors with low and moderate/high *LALBA* gene expression. A significant association was found between PD-L1 expression and *LALBA* levels, with higher PD-L1 expression observed in tumors exhibiting moderate-to-high LALBA expression compared with those showing low expression (*p* = 0.0402, two-tailed; Figure 6C). In contrast, no statistically significant differences were detected for PD-1, TIM-3 or LAG3 expression between the groups (Figure 6D–F). Given the number of immune markers analyzed and the absence of multiple-testing correction, this finding should be regarded as exploratory.

Finally, immune checkpoint coexpression patterns were examined, as the coexpression of inhibitory receptors—such as PD-1/TIM-3 and PD-1/LAG3—is commonly indicative of an exhausted T-cell phenotype. In this context, coexpression was evaluated descriptively as a phenotypic marker rather than as a functional or prognostic indicator. Accordingly, we evaluated the combined expression of these marker pairs and found no statistically significant differences between groups (PD-1/TIM-3: χ^2^ = 1.443, df = 3, *p* = 0.6955; PD-1/LAG3: χ^2^ = 2.392, df = 3, *p* = 0.4952; Figure 6G,H), indicating that these exhaustion-related coexpression patterns were not differentially associated with *LALBA* expression in this cohort.

**Figure 7:**
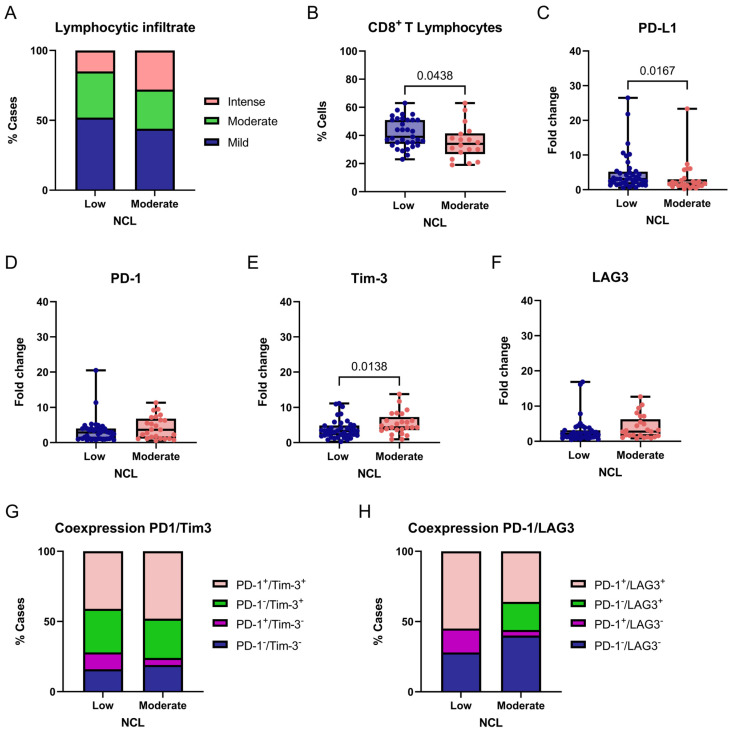
Association between *NCL* expression and immune-related markers in breast tumors. Comparison of degree of lymphocytic infiltration (**A**), percentage of CD8^+^ T cells (**B**), PD-L1 (**C**), PD-1 (**D**), Tim-3 (**E**), and LAG3 (**F**) expression levels, and PD-1/Tim-3 or PD-1/LAG3 coexpression (**G**,**H**) according to NCL expression groups (Low vs. Moderate/High). Box-and-whisker plot showing individual data points. Boxes represent the interquartile range (Q1–Q3) with the median line; whiskers extend to the minimum and maximum values. *n* = 65. Statistically significant differences were observed in the percentage of CD8^+^ T lymphocytes and PD-L1 and Tim-3 expression (Chi-square and Fisher’s exact tests or Mann–Whitney U test).

For *NCL*, we exploratorily compared tumors with low (fold-change values ≤ 1) and moderate (fold-change values > 1) gene expression levels in relation to immune-related markers in breast tumors. No adjustments for multiple comparisons were applied because this analysis was hypothesis-generating. No significant differences were observed in lymphocytic infiltration across *NCL* expression groups (χ^2^ = 5.007, df = 2, *p* = 0.0818; Figure 7A). However, a statistically significant difference was found in the proportion of CD8^+^ T cells within the lymphocytic infiltrate when comparing tumors with low and moderate *NCL* expression (*p* = 0.0438, two-tailed; Figure 7B). These findings suggest that *NCL* expression may not influence the overall level of lymphocytic infiltration but could be linked to differences in the proportion of CD8^+^ T cells within the infiltrate; however, the limited sample size and lack of functional immune profiling restrict biological interpretation.

We next evaluated the expression of the immune checkpoint markers PD-L1, PD-1, TIM-3, and LAG3 between tumors with low and moderate *NCL* gene expression. A significant association was found between PD-L1 expression and *NCL* levels, with higher PD-L1 expression observed in tumors exhibiting low NCL expression compared with those showing moderate expression (*p* = 0.0167, two-tailed; Figure 7C). Similarly, a significant association was detected for TIM-3, with lower TIM-3 expression observed in tumors exhibiting low NCL expression compared with those showing moderate expression (*p* = 0.0138, two-tailed; Figure 7E). In contrast, no statistically significant differences were found for PD-1 or LAG3 expression between the groups (Figure 7D,F).

Finally, considering PD-1 and TIM-3 coexpression, we found no significant differences between groups (χ^2^ = 3.843, df = 3, *p* = 0.2790; Figure 7G). In contrast, PD-1/LAG3 coexpression differed significantly according to *NCL* expression levels (χ^2^ = 34.13, df = 3, *p* < 0.0001; Figure 7H), suggesting a differential association of this exhaustion-related phenotype with *NCL* expression in this cohort. Post hoc analyses were performed using Fisher’s exact test. Tumors with low *NCL* expression showed a significantly higher frequency of PD-1^+^/LAG3^+^ double-positive cells compared with tumors exhibiting moderate *NCL* expression (*p* = 0.0104). This finding suggests that the observed association is primarily driven by an increased prevalence of this exhaustion-associated phenotype in the low *NCL* expression group. Therefore, this association should be interpreted as hypothesis-generating rather than as evidence of functional immune impairment.

## 3. Discussion

This study aimed to explore the expression of LALBA and NCL in breast cancer tissues from Mexican patients using gene expression analysis and immunohistochemistry, and to explore potential associations with clinicopathological characteristics, patient outcomes, and immune-related features within the constraints of an observational, cross-sectional design.

### 3.1. LALBA

As a key subunit of lactose synthase, LALBA is typically undetectable in most normal physiological context. However, consistent with its biological role, its expression increases markedly in the mammary gland during late pregnancy and lactation, when it plays an essential role in initiating and maintaining milk secretion [12]. In contrast to this expected pattern, we detected LALBA expression in nearly all analyzed tissues, both at the mRNA level and by immunohistochemistry. Our findings are consistent with those reported by Tuohy et al. (2016), who detected both LALBA gene and protein expression in 72.7% of Triple-Negative breast cancer biopsies [11]. Similarly, previous studies have reported LALBA protein expression by immunohistochemical analysis in 50.6% to 72.0% of breast cancer cases [17,18,19,20].

The upregulation of LALBA and other milk biosynthesis proteins in breast cancer patients has been linked to mutations in key regulatory genes, including BRCA1 [21], which encodes a protein essential for DNA repair, and PIK3CA [22], which encodes a protein involved in the regulation of critical cellular processes such as growth and cell division. Approximately 3–5% of breast cancer patients carry germline BRCA1 mutations, most commonly associated with the Triple-Negative subtype (65%). However, they have also been reported in 21% of Luminal B, 9% of Luminal A, and 6% of HER2+ cases [23]. In contrast, PIK3CA exhibits an overall somatic mutation rate of 26.7%, with higher frequencies reported in luminal subtypes (>30%), followed by 15.2% in HER2+ and 11.4% in Triple-Negative breast cancers [24].

Although no statistically significant association was observed between *LALBA* gene expression and tumor subtype, histological grade, clinical stage, or proliferation indices, *LALBA* overexpression was more frequently observed in tumors displaying aggressive features, particularly hormone receptor–negative status and high histological grade. Importantly, these features largely overlap with HER2-positive and triple-negative molecular subtypes, which represent major biological and clinical confounders. This observation led us to stratify patients into two groups: those with negative or low *LALBA* expression, and those with moderate-to-high expression, following a classification approach similar to that established for HER2 status in the ASCO–College of American Pathologists Guideline [25]. We found that patients with *LALBA* overexpression showed a trend toward shorter overall survival; however, this finding emerged from an unadjusted, exploratory analysis and is likely influenced by the underlying molecular subtype distribution rather than reflecting an independent prognostic effect of *LALBA* expression. Immunohistochemistry supported this interpretation, confirming that moderate LALBA expression was predominantly observed in the more aggressive cancer subtypes, namely HER2+ and Triple-Negative.

Our findings do not support LALBA as an independent prognostic biomarker, but rather suggest that its expression may serve as a surrogate marker of aggressive tumor phenotypes. Any potential prognostic or therapeutic relevance remains speculative and would require validation in larger, subtype-stratified cohorts and functional studies. Accordingly, further studies are necessary not only to validate these observations, but also to investigate the biological role of LALBA and the mechanisms driving its dysregulation in tumors. Future research should explore the relationship between *LALBA* expression and mutations in BRCA1 and PIK3CA, as well as the potential influence of parity. Notably, parity has been associated with increased expression of milk biosynthesis proteins, including LALBA, in patients with BRCA1 pathogenic variants [21]. A better understanding of how these genetic and reproductive factors interact to modulate *LALBA* expression, may help clarify whether LALBA has any indirect clinical relevance across breast cancer subtypes and provide valuable insights into its prognostic and therapeutic potential.

In relation to the inflammatory status of the tumor, the distribution of tumor-infiltrating lymphocytes (TILs) and PD-L1 expression is highly heterogeneous among breast cancer subtypes, with triple-negative and HER2-positive tumors generally exhibiting greater immune cell infiltration and PD-L1 expression than luminal-like tumors [26]. The overexpression of HER2 can actively engage the immune system by functioning as a tumor-associated antigen, as HER2-derived peptides are presented by major histocompatibility complex (MHC) molecules, leading to cytotoxic T lymphocyte recognition and activation of an antitumor immune response. This immune engagement contributes to the elevated TILs levels typically observed in HER2-positive tumors and can also induce the upregulation of immune checkpoint molecules such as PD-L1, facilitating immune evasion. Therefore, HER2 overexpression not only drives tumor aggressiveness but also modulates the tumor immune microenvironment, influencing prognosis and response to both HER2-targeted and immunotherapeutic treatments [27].

Building on the link between tumor aggressiveness and immune engagement in HER2-positive tumors, we investigated whether markers such as LALBA and NCL might be associated with immune features in tumors. Tumors with moderate-to-high LALBA expression showed increased lymphocytic infiltration and higher PD-L1 levels, a pattern that parallels the enrichment of aggressive molecular subtypes known to display greater immune activity. However, no significant differences were observed in CD8^+^ T-cell infiltration or in the expression of PD-1, TIM-3, or LAG3. Collectively, these findings do not support a direct association between elevated LALBA expression and an enhanced antitumor immune response, nor do they allow inferences regarding prognosis or treatment response. Rather, the observed immune-related features are most plausibly attributable to the underlying molecular subtype context.

### 3.2. Nucleolin

NCL is a highly conserved, multifunctional phosphoprotein that participates in diverse cellular processes, including gene silencing, DNA and RNA metabolism, chromatin remodeling, and microtubule organization. Through these functions, NCL plays a central role in sustaining cell viability, regulating proliferation, and orchestrating responses to cellular stress. Although it is broadly distributed across the plasma membrane, cytoplasm, and nucleoplasm, the majority of NCL—approximately 90%—is localized within the nucleolus. This dynamic subcellular distribution is critical, as the localization of NCL strongly influences both its biological functions and its contributions to pathological states [28].

Since no clear association was observed between *NCL* gene expression and tumor subtype, histological grade, clinical stage, or proliferation indices, we used the same patient stratification approach previously applied for *LALBA*. Interestingly, we found that patients with lower levels of *NCL* expression had significantly poorer overall survival compared to those with moderate *NCL* mRNA expression. These results are in line with the findings of Nguyen Van Long et al. (2018), who also described an association between intermediate *NCL* mRNA levels and increased overall survival [29]. Moreover, they observed that tumors with low *NCL* expression exhibited mRNA levels similar to those in normal mastectomy tissues, further suggesting that both under- and overexpression of *NCL* may reflect distinct biological states with prognostic significance.

In our study, IHC analysis revealed NCL expression confined exclusively to the nuclear compartment of tumor cells. The absence of cytoplasmic or membrane-associated staining may be consistent with a more regulated or less transformed cellular state; however, this interpretation remains speculative, as subcellular localization was not functionally interrogated. Nevertheless, in cancer cells, enzymatic modifications may drive the spontaneous translocation of NCL from the nucleus to the cytoplasm, thereby contributing to tumor progression. This is particularly relevant since NCL redistribution to the cytoplasm and cell surface has been associated with adverse clinical outcomes, including increased lymph node metastasis, reduced survival, and higher expression in poorly differentiated tumors [30,31].

Based on the prior literature, activation of the phosphoinositide 3-kinase (PI3K) signaling cascade by growth factors, particularly vascular endothelial growth factor (VEGF), is known to promote the translocation and stabilization of NCL at the cell surface. Once localized to the plasma membrane, NCL interacts with various extracellular ligands to modulate critical biological processes. These include cell differentiation, adhesion, leukocyte trafficking, inflammatory responses, angiogenesis, and tumor progression [28]. These mechanisms were not assessed in the present study and are discussed here solely as a biological context, rather than as direct explanations of our findings.

Together, these data highlight the complex and context-dependent role of NCL in breast cancer biology, supporting its potential utility as a prognostic biomarker. However, further studies are necessary to investigate the mechanisms driving NCL dysregulation in tumors and the precise mechanisms driving its surface localization. Future research should investigate the relationship between NCL expression and VEGF context in tumors, as well as the impact of PIK3CA mutations.

In relation to the inflammatory status, immune-high tumors are typically characterized by strong TIL’s presence and elevated PD-L1 expression, a profile commonly associated with triple-negative and HER2-positive subtypes. In contrast, hormone receptor positive tumors usually exhibit limited immune cell infiltration and tend to be unresponsive to immunotherapy [26]. Therefore, the reduced CD8^+^ T cell presence and lower PD-L1 expression observed in tumors with higher NCL expression could reflect a less immunogenic phenotype, more typical of luminal-like breast cancers, and it is consistent with our results.

Interestingly, unlike ovarian and lung cancers, the presence of TIM-3 on TILs in breast cancer has been correlated with improved survival and may therefore represent a favorable prognostic factor [32]. In line with this, we observed higher TIM-3 expression in tumors with moderate *NCL* expression. No significant differences were observed in PD-1 or LAG3 expression, nor PD-1/TIM-3 coexpression; however, a significant difference in PD-1/LAG3 patterns was observed according to NCL levels, with a higher frequency of PD-1^+^/LAG3^+^ double-positive cells in tumors exhibiting low *NCL* expression.

Given that PD-1/LAG3 coexpression is commonly used as a phenotypic marker associated with T-cell exhaustion, this observation may suggest a less effective antitumor immune environment in tumors with low *NCL* expression; however, this observation should be interpreted cautiously, as no functional immune assays were performed to confirm T-cell dysfunction or immune exhaustion.

Therefore, although elevated *NCL* expression cannot be directly linked to antitumor immune activity or treatment response in this study, the immune checkpoint expression patterns observed are compatible with a more favorable immune-related prognosis in tumors with moderate *NCL* expression.

## 4. Materials and Methods

### 4.1. Study Design and Participants

We conducted a cross-sectional study that included 65 Mexican women with a clinically and histopathological confirmed diagnosis of breast cancer. Those patients were recruited at the University of Guadalajara as part of the “ELLA Binational Breast Cancer Study”, a multicenter study aimed at identifying molecular markers of breast tumors, clinicopathological characteristics, and risk factors in women [33]. Their clinical features, molecular phenotype, and clinical and pathological stage were obtained through medical records. The study was approved by the Ethics, Research, and Biosafety Committee of the University of Guadalajara, and all women provided informed consent.

### 4.2. Gene Expression

Total RNA was extracted from 65 biopsies or surgical resections of breast cancer tissue, depending on sample availability for each patient. The pathologist confirmed the presence of sufficient tumor cells in the sample used for microarray analysis, which contained more than 60% tumor cells and less than 20% necrosis, as determined by hematoxylin-eosin (H&E)-stained slides. RNA quantity and purity were assessed using the NanoDrop (Wilmington, DE, USA), and only samples with a RNA integrity number (RIN) ≥ 4 were included in the study (more than 90% of the patient samples had RIN ≥ 6). For gene expression analysis, the Human Gene Expression 4 × 44K v2 Microarray Kit (G2519F-026652, Agilent Technologies, Santa Clara, CA, USA) was used. Universal Reference RNA (Stratagene, La Jolla, CA, USA) served as the control. In this procedure, RNA from each experimental sample and the Universal Reference RNA were labeled with distinct fluorophores and co-hybridized onto the same chip. Data acquisition and processing were performed using Agilent’s Feature-Extraction software (https://www.agilent.com.cn/zh-cn/product/mirna-microarray-platform/mirna-microarray-software/feature-extraction-software-228496, accessed on 30 May 2025), which calculates fold-changes between experimental and reference samples using Equation (1):

(1) $$Fold-change=2^{log2(experimental\;fluorescence\;intensity/reference\;fluorescence\;intensity)}$$

A fold-change value of 1 corresponds to basal gene expression relative to the reference sample. Values greater than 1 indicate relative overexpression, whereas values between 0 and 1 indicate relative underexpression. This threshold corresponds to the biologically defined basal expression level and coincides with the cohort median for both genes, supporting its use as a data-driven and non-arbitrary cutoff.

Quality control included spike-in controls evaluation, intensity distribution, background uniformity, signal-to-noise ratio (SNR), and artifacts. Arrays with r < 0.99 for spike-ins, SNR < 2, or spatial defects were removed. Background correction was performed. For microarray normalization, we used Agilent’s Two-Color Microarray-based Gene Expression Analysis, which uses cyanine 3- and cyanine 5-labeled targets to measure gene expression in experimental and control samples. We used a Stratagene universal reference RNA (La Jolla, CA, USA) as a quality control for the samples. Additionally, as part of data normalization, Agilent provides proprietary software (Agilent Feature Extraction) for image analysis and pre-processing of microarray data. This software helps extract relevant features from images and generate initial, normalized data for further analysis.

### 4.3. Automated Immunohistochemistry

A total of 51 formalin-fixed paraffin-embedded breast cancer tissue samples derived from the “ELLA Binational Breast Cancer Study” were included in the study. Tissue sections of 3 μm were prepared and mounted on hydrophilic adhesion slides (Cat. No. TOM-11 White 25 × 75 × 1.0 mm; Matsunami, Kishiwada City, Osaka, Japan), then deparaffinized and rehydrated. The samples were processed automatically in the BOND-MAX equipment (Leica Biosystems, Deer Park, IL, USA). Immunodetection was performed according to the manufacturer’s guidelines for the Bond polymer refine detection system (DS9800, Leica Biosystems, Deer Park, IL, USA). Antigen recovery was performed with EDTA buffer solution (pH 8.0) for LALBA or citrate buffer solution (pH 6.0) for NCL. The primary antibodies used were LALBA (Cat. No. HPA029856, Sigma-Aldrich, St. Louis, MO, USA; 1:200) and nucleolin (Cat. No. ab136649, Abcam, Waltham, MA, USA; 1:100). Normal breast and amygdala tissues served as a positive controls. A board-certified pathologist, blinded to the molecular classification and clinical characteristics of the cases, performed a semiquantitative visual analysis of the IHC assays. At 4× magnification, the tumor area was first identified, and subsequently analyzed at 40× magnification. The pathologist assessed the presence (positive/negative), intensity (defined as 1+ weak, 2+ moderate, and 3+ strong staining), and staining pattern of each marker. Cases were considered positive when ≥5% of tumor cells exhibited cytoplasmic and/or nuclear staining at any intensity level; scores were used for exploratory and comparative analyses. Finally, slides were scanned and digitized with the Aperio LV1 real-time pathology system (Leica Biosystems, Deer Park, IL, USA).

### 4.4. Statistical Analysis

To assess the normality of the data distribution, the Shapiro–Wilk test was performed. Because the data did not follow a normal distribution, the nonparametric Kruskal–Wallis test was used to analyze LALBA and NCL gene expression levels across tumor samples. Kaplan–Meier and log-rank analyses were used to analyze survival curves. A univariate Cox proportional hazards model was used to examine the association between moderate/high versus low expression levels of LALBA and NCL. Patients with fold-change values ≤ 1 were classified as having low expression, those with values between 1 and 5 as having moderate expression, and those with values > 5 as having high expression. These thresholds were selected to enable stratification of samples into biologically interpretable expression categories for exploratory analysis, rather than to define clinically validated cutoffs.

For immunohistochemistry analysis, expression levels were grouped into two categories—No/Low and Moderate/High—a dichotomization applied to increase statistical robustness given the sample size and the semi-quantitative nature of the scoring system. The association between marker expression and breast cancer subtypes, lymphocytic infiltrate or coexpression of PD-1/Tim-3, was assessed using the Chi-square (χ^2^) test for independence. Post hoc pairwise comparisons were performed using Fisher’s exact test. The Mann–Whitney U test was applied to compare the expression levels of PD-L1, PD-1, Tim-3, and the percentage of CD8^+^ T cells between groups with low versus moderate/high LALBA or NCL expression.

Statistical analyses were conducted using GraphPad Prism version 9 (GraphPad Software, San Diego, CA, USA), and a *p*-value  <  0.05 was considered statistically significant. All analyses were performed without multivariate adjustment and should be interpreted as exploratory.

## 5. Conclusions

Our findings suggest that NCL and LALBA are associated with distinct features of breast tumor biology, based on exploratory analyses in a limited cohort. Higher LALBA expression was observed in aggressive tumors and was associated with increased lymphocytic infiltration and elevated PD-L1 levels, indicating a possible relationship with tumor aggressiveness and immune-related features. However, since no significant differences were observed in CD8^+^ T cells, PD-1, or TIM-3, the direct impact of LALBA on antitumor immunity, prognosis, or treatment response remains uncertain. In contrast, while abnormal NCL redistribution has been linked to cancer progression and poor prognosis in other tumor types, our observations indicate that, in this cohort, NCL expression was predominantly restricted to the nucleus and was associated with reduced CD8^+^ T cell infiltration, lower PD-L1 levels, and elevated TIM-3 expression. This pattern may reflect a less immunogenic phenotype, more typical of luminal-like breast cancers, without implying independent prognostic significance. These findings underscore the need for larger, well-characterized cohorts to clarify the molecular mechanisms underlying LALBA and NCL dysregulation, their influence on the tumor immune microenvironment, and their potential as therapeutic targets across different breast cancer subtypes.

## 6. Limitations

This study has several limitations that should be considered when interpreting the results. First, the sample size was limited, especially in immunohistochemical analysis, which may affect the generalizability of our findings across different breast cancer subtypes. In addition, the limited cohort size precluded the use of multivariate models adjusted for major clinical and pathological confounders, and no correction for multiple testing was applied. Therefore, the reported associations should be interpreted cautiously, as they may be susceptible to type I error and overestimation of effect sizes.

Second, protein localization and expression do not directly address the underlying functional mechanisms or post-translational modifications that may influence NCL and LALBA activity. Third, the cross-sectional design prevents establishing causal relationships between protein expression, genetic alterations, and clinical outcomes. Furthermore, detailed information regarding treatment regimens and standardized follow-up durations was not available for all patients; consequently, survival analyses could not be adjusted for treatment-related variables or therapy response, and cohort heterogeneity in clinical management may have influenced the observed survival and immune-related associations.

Importantly, molecular subtype is a dominant confounder in breast cancer biology; however, subtype-adjusted analyses were limited by sample size and therefore could not be comprehensively performed. Finally, factors such as parity and specific mutations (e.g., BRCA1, PIK3CA) were not comprehensively controlled, which could influence LALBA and NCL expression and its prognostic relevance.

Future studies with larger, well-characterized cohorts, combined with mechanistic and functional analyses, are warranted to validate these findings and further elucidate the roles of NCL and LALBA in tumor progression.

## Figures and Tables

**Table 1:** Immunophenotypically definitions of molecular groups of invasive breast carcinoma subtypes.

Marker	Luminal A	Luminal B (HER2−)	Luminal B (HER2+)	HER2-Enriched	Triple-Negative
ER	+	+	+	−	−
PR	+	−/low	any	−	−
HER2	−	−	+	+	−
Ki-67	low	high	any	any	Any

**Table 2:** Clinical and pathological characteristics of the patients included in this study.

Molecular Subtype	Luminal A	Luminal B	HER2-Enriched	Triple-Negative
Number of patients	21	14	12	18
Age range (*n*)				
Unknown	1	0	0	0
<40	0	2	1	7
40 to 50	5	3	2	6
51 to 70	11	8	9	4
Histologic type (*n*)				
Invasive ductal carcinoma	19	8	10	12
Invasive lobular carcinoma	1	5	0	0
Invasive mixed ductal and lobular carcinoma	1	1	0	1
Others	0	0	2	5
Histological grade (*n*)				
Low	7	1	0	1
Intermediate	12	12	7	5
High	2	1	5	12
Clinical stage (*n*)				
Stage I	1	0	2	1
Stage II	9	8	1	10
Stage III	8	5	8	4
Stage IV	3	1	1	3
Ki67 (*n*)				
Low risk (≤14%)	20	0	1	6
Intermediate risk (15–29%)	1	6	2	4
High risk (≥30%)	0	8	9	8

Data are expressed as absolute frequencies (*n*).

## Data Availability

The original contributions presented in this study are included in the article. Further inquiries can be directed to the corresponding authors.
